# Application of 3D reconstruction and 3D printing technology in advanced ovarian cancer surgery: a retrospective study

**DOI:** 10.3389/fonc.2024.1432970

**Published:** 2024-08-16

**Authors:** Zhihui Cai, Ke Zhang, Linqian Li, Yuping Suo

**Affiliations:** ^1^ Gynecology Department, The Fifth Clinical Medical School of Shanxi Medical University, Taiyuan, Shanxi, China; ^2^ 3D Image and 3D Printing Center, Affiliated Hospital of Hebei University, Baoding, Hebei, China

**Keywords:** ovarian cancer, 3D visualization, 3D printing, preoperative planning, intraoperative risks

## Abstract

**Backgrounds:**

Advanced ovarian cancer is frequently accompanied by extensive peritoneal metastasis, complicating surgical interventions. This study aims to explore the application of 3D reconstruction and 3D printing technology in the treatment of advanced ovarian cancer.

**Methods:**

We conducted a retrospective analysis of 60 patients with stage III ovarian cancer who underwent cytoreductive surgery at Hebei University Affiliated Hospital between 2020 and 2023. Patients were randomly assigned to three groups: a 3D visualization group, a 3D visualization plus 3D printing group, and a traditional 2D CT imaging evaluation group. High-precision medical imaging techniques (e.g., CT, MRI) were employed to create digital 3D models, which were then converted into physical entities using 3D printing for surgical planning and simulation.

**Results:**

Both the 3D visualization group and the 3D visualization plus 3D printing group demonstrated superior outcomes in terms of surgery duration and blood loss compared to the traditional 2D CT group, indicating the efficacy of 3D reconstruction and 3D printing in preoperative planning. Postoperative recovery indicators, such as hospital stay and time to first flatus, were also more favorable in the groups utilizing 3D technology. Although there were no significant differences in postoperative complications and recurrence rates among the three groups, the groups using 3D technology showed advantages in reducing certain complications.

**Conclusions:**

The results indicate that medical 3D technology has significant value in the surgical planning of advanced ovarian cancer, enhancing surgical precision and reducing intraoperative risks, which may aid in improving postoperative recovery.

## 1 Introduction

Ovarian cancer is a leading malignancy in the female reproductive system, and it has become the primary cause of death among gynecological cancers in China. Over the past decade, its incidence has increased by 30%, with a corresponding 18% rise in mortality (1). A major challenge in ovarian cancer is its insidious progression, often leading to extensive peritoneal metastasis by the time of diagnosis, which complicates surgical interventions. Studies indicate that the five-year survival rate for patients with advanced ovarian cancer is only 25% (2). Prognosis in ovarian cancer patients is closely related to the size of the residual tumor post-surgery, making maximal tumor resection crucial. In addition to conventional total hysterectomy and bilateral salpingo-oophorectomy, advanced ovarian cancer treatment may require more complex surgeries such as bowel resection, partial gastrectomy, diaphragm or other peritoneal stripping, splenectomy, partial hepatectomy, cholecystectomy, partial cystectomy, ureteral resection, and distal pancreatectomy, which are associated with significant trauma, bleeding, and risk of complications (3, 4).

The integration of medical 3D reconstruction and 3D printing technology represents a major innovation in the healthcare sector, providing new avenues to improve diagnostic and therapeutic accuracy and efficiency. This process relies on high-precision medical imaging data (e.g., CT, MRI, or ultrasound) that are reconstructed into digital 3D models. These models are then transformed into physical entities through layer-by-layer printing, accurately replicating internal human structures (5, 6). Medical 3D digital models and printed physical models have extensive applications in surgical planning, simulation, patient education, and the design of customized medical devices and implants. They enhance surgical accuracy and safety, offering personalized medical solutions to patients. In complex tumor resections or organ transplantation surgeries, these models help surgeons understand specific anatomical details in advance, optimizing surgical paths and reducing intraoperative uncertainties.

The primary objective of this study is to explore the application and potential benefits of 3D reconstruction and 3D printing technology in the treatment of advanced ovarian cancer, focusing on how this technology can enhance surgical precision and reduce the likelihood of surgical complications. Through an in-depth study of this emerging technology, we aim to provide more effective methods and insights for improving treatment outcomes for patients with advanced ovarian cancer.

## 2 Clinical data and methods

### 2.1 General data and ethical approval

In this retrospective study, we analyzed 60 patients with stage III ovarian cancer who underwent cytoreductive surgery at Hebei University Affiliated Hospital between 2020 and 2023. To evaluate the impact of different assessment methods on surgical outcomes, participants were randomly assigned to three groups: Group A (3D visualization group), Group B (3D visualization plus 3D printing group), and Group C (traditional 2D CT imaging evaluation group).

**Inclusion criteria:**

1. Diagnosed with stage III ovarian cancer.
2. Age between 18 and 70 years.
3. No contraindications to surgery.
4. No prior cancer-related treatments (e.g., chemotherapy, radiotherapy).
5. Complete medical records and imaging data.
6. Signed informed consent by the patient or legal guardian.
7. No neoadjuvant chemotherapy before surgery.
8. No need to preserve fertility.

**Exclusion criteria:**

1. Severe dysfunction of major organs such as heart, liver, or kidneys.
2. Other types of malignancies.
3. Pregnant or lactating women.
4. Preoperative neoadjuvant chemotherapy for ovarian cancer.
5. Need to preserve fertility.
6. Other cancer treatments within six months before the study.
7. Inability to complete the study.

We adhered to strict ethical standards in this study. All participants read and signed detailed informed consent forms before joining the study. These forms covered the study’s purpose, procedures, potential risks and benefits, and privacy protection measures. We ensured voluntary participation for all, emphasizing their right to withdraw consent at any time without affecting the medical care they received at Hebei University Affiliated Hospital. This study was approved by the Hebei University Affiliated Hospital Ethics Committee, ensuring compliance with ethical review standards.

### 2.2 Preoperative planning

**3D visualization group (Group A):** The preoperative planning process involved using enhanced CT (Revolution CT, GE Healthcare, Milwaukee, USA) to complete a full scan from the diaphragm to the pubic symphysis in one breath-hold. The scan phases included arterial, venous, and excretory phases. During the enhanced scan, 80 mL of iodinated contrast agent and 50 mL of saline were intravenously injected at a rate of 3.5 mL/s. Arterial phase scanning time was 25 seconds, venous phase 65 seconds, and excretory phase 300 seconds. The collected CT images were imported in DICOM format into MIMICS 23.0 software (Materialise NV, Leuven, Belgium), where window width and level were adjusted for optimal image reconstruction. The CT Bone function was used for automatic segmentation and reconstruction of the pelvis and sacrum. Then, a new mask was created, and region grow, split mask, smooth mask, Boolean operations, and calculate part steps were executed to construct a 3D ovarian cancer model, including organs like the uterus, pelvic arteries and veins, lymph nodes, bladder, ovaries, ascites, and colorectal segments, each set in different colors.

The generated STL files were further processed in 3-matics 15.0 software (Materialise NV, Leuven, Belgium) for wrapping, adaptive remeshing, hole filling, smoothing, and other post-processing steps. The files were then imported into E3D digital medical modeling and design software (Central South University, China) and uploaded to a cloud platform. The generated link allowed doctors to interact with the models on a computer or mobile device using mouse or gesture controls.

**3D visualization plus 3D printing group (Group B):** After obtaining the 3D reconstruction data using the previously described method, pre-printing processing involved checking model integrity, repairing holes, adjusting model size to meet printing requirements, and setting printing parameters (layer height, density, temperature, etc.). The processed models were imported into an SLS (Yingpu, China) 3D printer. Flexible and elastic TPU material was used for arteries, veins, and tumors, while high-strength, wear-resistant nylon material was used for bones. The final ovarian tumor model was created using a combination of rigid and flexible printing methods. Post-processing steps included cooling, powder removal and recovery, surface treatment, and dyeing. Detailed preoperative assessment and precise surgical planning were conducted based on these 3D images and printed models. This process allowed for a comprehensive understanding of complex tumor structures, optimizing surgical plans and increasing the success rate.

**Traditional 2D CT imaging evaluation group (Group C):** Participants’ preoperative assessment relied entirely on traditional 2D CT scanning technology. Transverse images of the pelvic area were obtained using 2D CT, assessing tumor location, size, and its relationship with surrounding structures. These images were interpreted to determine tumor location, potential invasion of adjacent organs or structures, and surgical resectability and planning, mitigating intraoperative bleeding risks.

### 2.3 Surgical procedure

All surgeries were performed by the same experienced surgical team. For patient safety and comfort, all surgeries were conducted under general anesthesia. The core steps included a paramedian incision into the abdomen, total hysterectomy, bilateral salpingo-oophorectomy, and omentectomy. For tumors invading adjacent organs or peritoneum, the team performed combined organ or peritoneal resections as needed, aiming to remove as much tumor tissue as possible while preserving the function of surrounding healthy tissues and organs. Postoperative drainage tubes were placed in the pelvis or abdomen. Preventive anti-infection and appropriate fluid therapies were provided to support postoperative recovery. The entire surgical and postoperative care process aimed to optimize treatment outcomes, reduce patient discomfort, and promote rapid recovery.

### 2.4 Observation and evaluation indicators

A series of detailed evaluation indicators were adopted to comprehensively assess the surgical process and its impact on patient recovery. Intraoperative indicators included blood loss, surgery duration, intraoperative complications, and transfusion volume, reflecting surgical safety and complexity. Short-term postoperative recovery indicators included hospital stay, time to first flatus, postoperative complications, blood test results on the second postoperative day, time to first ambulation, days with pelvic drainage tube, and days of postoperative fever, evaluating patient recovery speed and smoothness. Tumor recurrence time was used as a long-term follow-up indicator to assess the lasting effect of surgical treatment.

### 2.5 Statistical methods

To evaluate and compare differences in intraoperative and postoperative indicators among groups A, B, and C, statistical analysis was performed using SPSS Statistics software. One-way ANOVA was used to compare mean values across three or more independent sample groups. A P-value of less than 0.05 was considered statistically significant, indicating significant differences in corresponding indicators among different groups.

## 3 Results

### 3.1 General clinical information

Sixty patients with stage III ovarian cancer were evenly divided into three different treatment groups: Group A, Group B, and Group C, each with 20 patients. Regarding ovarian cancer staging, there were 5 patients in stage IIIA, 13 in stage IIIB, and 42 in stage IIIC. In terms of surgical methods, 33 patients underwent R0 surgery, 23 underwent R=1 surgery, and 4 underwent R>1 surgery. There were no significant differences in age, BMI, pathological grouping, or surgical methods among the groups, indicating balanced baseline data (see **Table 1**).

**Table 1:** General information statistics of patients.

Parameter		Group A	Group B	Group C	χ2/F	P
**Age (years)**		57.15 ± 10.65	54.60 ± 8.56	58.60 ± 10.16	0.976	0.433
**BMI**		24.83 ± 2.91	25.06 ± 3.88	25.44 ± 2.66	0.186	0.831
**Pathological Stage**	IIIA	2	1	2	1.236	0.872
	IIIB	3	5	5		
	IIIC	15	14	13		
**Surgical Method**	RO	10	11	12	0.742	0,946
	R=1	8	8	7		
	R>1	2	1	1		

The values for Age and BMI are mean ± standard deviation. The Pathological Stage and Surgical Method sections include counts of patients within each category.”χ2/F” and “P” columns likely represent statistical measures, with “χ2/F” being the Chi-square or F-value and “P” for the p-value in statistical testing.

### 3.2 Comparison of perioperative indicators

In terms of intraoperative conditions, we observed significant differences in surgery duration, blood loss, and transfusion volume among the three groups (P < 0.05, see **Table 2**). Groups A and B had shorter surgery durations and less blood loss compared to Group C, with statistically significant differences. However, no significant differences were observed between Groups A and B, although Group B performed better in most indicators.

**Table 2:** Comparison of perioperative indicators of patients.

Parameter	Group A (n=20)	Group B (n=20)	Group C (n=20)	χ2/F	p
Op. Time (min)	393.25 ± 41.40	390.75 ± 30.62	428.25.07 ± 43.69*#	5.784	0.005
Blood Loss (cc)	706.00.33 ± 207.30	669.00 ± 204.63	898.00 ± 222.04*#	6.759	0.002
Blood Transfusion (cc)	0 (300)	0 (350)	400 (400)*#	6.440	0.040
Post-op Day 2 WBC (x10^9/L)	11.22 ± 1.75	10.57 ± 2.32	13.17 ± 2.42*#	7.688	0.001
Post-op Day 2 Hb (g/L)	97.30 ± 6.16	97.90 ± 6.60	95.90 ± 6.37	0.518	0.599
Time to Flatus (days)	3.0 (1.0)	3.0 (1.0)	5.0 (1.0)*#	21.970	0.000
Time to Ambulation (days)	4.0 (1.0)	4.0 (1.0)	5.0 (1.0)*#	22.365	0.000
Drainage Duration (days)	5.0 (1.0)	5.0 (1.0)	6.0 (2.0)*#	12.098	0.002
Fever Duration (days)	0.0 (3.0)	0.0 (3.0)	0.0 (3.0)	1.611	0.447
Intra-op Complications	/	/	/		

The “*” indicates statistical significance compared to Groups A and B, while “#” indicates significance compared to Group B only.

Op. Time, Operation Time; WBC, White Blood Cells; Hb, Hemoglobin; Post-op, Postoperative.

Intra-op: Intraoperative;cc: Cubic Centimeters (also a measure of volume, equivalent to milliliters).

In postoperative recovery, significant differences in time to first flatus, time to first ambulation, and duration of drainage tube placement were observed among the three groups (P < 0.05). Groups A and B showed notably shorter recovery times compared to Group C.

There were no significant differences in postoperative fever duration and hemoglobin levels on the second postoperative day among the three groups (P > 0.05).

### 3.3 Comparison of postoperative complications and prognosis

We analyzed the impact of different preoperative planning methods on surgical outcomes. Statistical results showed no significant differences in postoperative complications and recurrence rates among the three groups (chi-square test, P > 0.05, see **Table 3**).

**Table 3:** Postoperative complications and recurrence.

Parameter		Group A	Group B	Group C	χ2/F	P
Post-op Complications	Hypoalbuminemia	5	7	11	1.357	0.507
	Pulmonary Infection	3	2	7	2.184	0.335
	Wound Dehiscence	2	2	5	1.576	0.455
	Bacteremia	0	0	1	0.155	0.926
	LLVT	3	3	6	0.387	0.824
Post-op Recurrence		5	5	7	0.155	0.926

χ2/F” and “P” are statistical measures, with “χ2/F” possibly indicating Chi-square or F-value, and “P” for the p-value.

Post-op, Postoperative; LLVT, Lower Limb Venous Thrombosis.

### 3.4 Case studies

**Figure 1:**
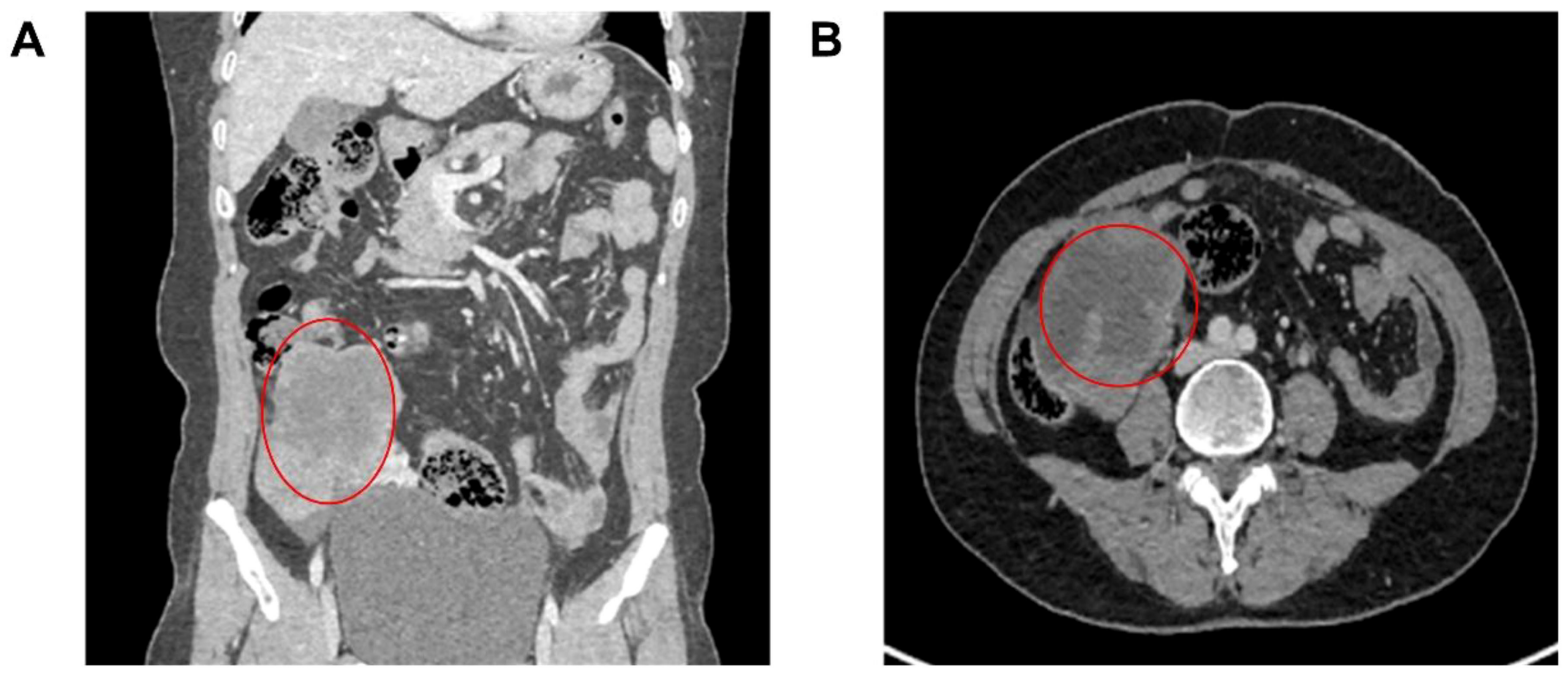
Patient’s CT images. **(A)** CT showed a solid cystic mass in the right adnexal area; **(B)** Enhanced CT indicated a suspicious malignant mass in the right adnexal area, and enlarged retroperitoneal lymph nodes. The marked location was a space-occupying lesion in the right adnexal area, with a high possibility of malignancy

1. **Patient A:** A 57-year-old female presented with lower abdominal distention and pain for 8 days. Gynecological examination revealed a cystic-solid mass in the right anterior uterus, with poor mobility and close relation to the uterus. Transvaginal ultrasound indicated a cystic-solid mass in the right adnexal area, approximately 10.3x7.4x6.4 cm in size, with clear boundaries, irregular shape, internal blood flow signals, and surrounding fluid dark areas. Enhanced CT of the abdomen and pelvis indicated a hepatic cyst in the right lobe, a suspicious malignant mass in the right adnexal area, and enlarged retroperitoneal lymph nodes (See **Figure 1**). Tumor marker CA125 was elevated at 209.7 U/mL.

**Surgery:** The surgery revealed a cystic-solid mass in the right adnexal area, approximately

10 cm in diameter, closely adhered to the uterus, right pelvic wall, sigmoid colon, and rectum, with the pelvis completely closed. After gradually separating the adhesions, the right ureter was freed, and the right adnexa were removed. Intraoperative frozen sections suggested a high-grade serous carcinoma of the right ovary. The surgery included total hysterectomy, left adnexectomy, omentectomy, pelvic metastasis resection, and resection of enlarged pelvic and abdominal lymph nodes. Preoperative assessment and surgical planning ensured a smooth surgery, lasting 4 hours and 20 minutes, with approximately 480 mL of blood loss. The postoperative pathology confirmed high-grade serous carcinoma of the right ovary. The patient recovered well and was discharged on the 8th day post-surgery without any intraoperative or postoperative complications.

2. **Patient B:** A 67-year-old female presented with persistent abdominal distention for one year. Abdominal examination revealed significant abdominal distension and positive shifting dullness. Gynecological examination indicated a cystic-solid mass in the right adnexal area, approximately 15 cm in diameter, closely connected to the uterus with poor mobility. Transvaginal ultrasound showed a cystic-solid mass in the pelvis, approximately 15x7.1 cm. Enhanced CT of the abdomen and pelvis indicated a large irregular cystic-solid mass in the pelvis, approximately 13x11x8 cm, with unclear boundaries and complex internal structure. Blood tumor markers showed elevated levels of CEA (17.6 ng/mL), CA125 (107 U/mL), and CA199 (7062 U/mL).

**Figure 2:**
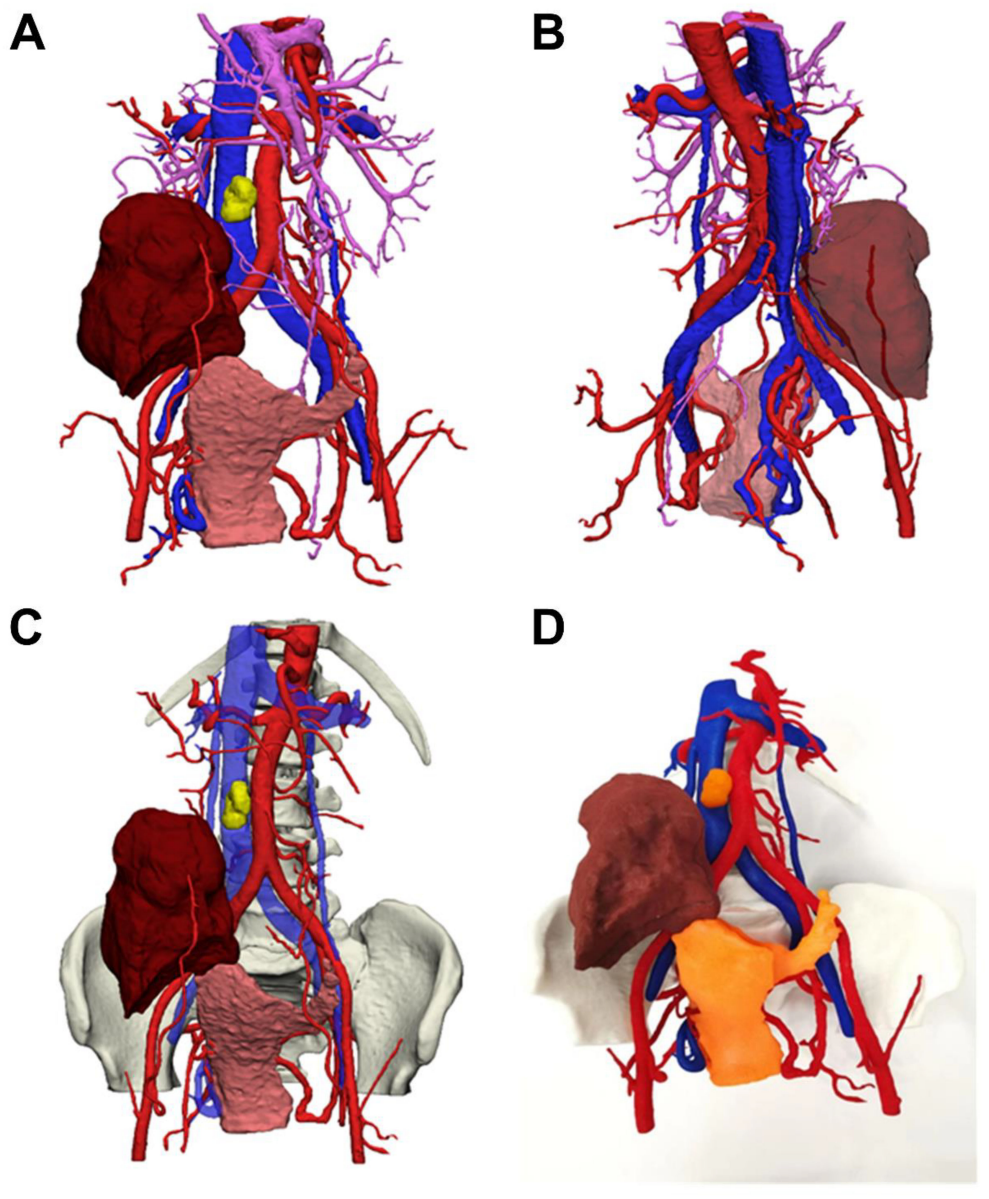
Patient 3D reconstruction and 3D printed model **(A)** Main view of 3D modeling; **(B)** Posterior view of 3D modeling; **(C)** Main view of 3D modeling (including pelvic structures); **(D)** 3D printed solid figure; Note: Blue is inferior vena cava, red is abdominal aorta, purple is superior mesenteric vein; yellow is lymph node; brown is: ovarian tumor; pink is uterus (shown in orange in 3D model).

**Surgery:** Preoperative 3D reconstruction indicated that the tumor’s arterial blood supply originated mainly from three branches of the left ovarian artery and a smaller branch of the iliac vessels (see **Figure 2**). The venous system was more complex, with two large venous branches from the right internal iliac vein collecting blood from the tumor’s right posterior side, indicating careful ligation was necessary to reduce bleeding risk. Surgery revealed dense adhesions between the tumor and surrounding tissues, increasing the difficulty of separation. Guided by 3D reconstruction images, the right peritoneum was incised first to identify the right ureter, followed by the careful separation of the right ovarian vessels. The surgery included total abdominal hysterectomy, omentectomy, pelvic and abdominal metastasis resection, appendectomy, and resection of enlarged lymph nodes, with a surgery duration of 3.5 hours and blood loss controlled to within 400 mL. The patient recovered well and was discharged on the 10th day post-surgery without any intraoperative or postoperative complications.

## 4 Discussion

The complexity of advanced ovarian cancer surgery and its impact on patient prognosis is a focal point in medical research. In advanced cases, tumors often spread to multiple surrounding organs and tissues, making complete surgical resection extremely challenging. Studies have shown that the thoroughness of surgery is crucial in improving patient prognosis. Effective cytoreductive surgery can significantly increase survival rates, demanding higher levels of surgical skill and precision. Particularly for stage III ovarian cancer patients, satisfactory cytoreductive surgery is essential for prognosis and survival. Research indicates that for every 10% increase in the satisfaction of cytoreductive surgery, median survival time can be extended by 5.5 months (7, 8). Additionally, this surgery reduces tumor burden to a minimum, enhances sensitivity to chemotherapy and radiotherapy, improves immune response, and alleviates symptoms. For initial treatment patients, satisfactory cytoreduction with no visible residual tumor has become the surgical goal. Achieving this often requires extensive tissue removal and combined organ resection, posing high surgical difficulty and risk of intraoperative complications (9).

This study focuses on the application of 3D reconstruction and 3D printing technology during the surgical process. These advanced technologies offer unprecedented precision in surgery. By transforming detailed medical imaging data into 3D digital models, surgeons can gain deeper insights into tumor specifics and their relationships with surrounding vital structures.

In clinical practice, the application of 3D reconstruction and 3D printing technology has shown immense potential and success across various medical fields, playing a crucial role in preoperative planning and preparation. Creating patient-specific 3D models enables more accurate understanding of patient anatomy, facilitating more precise surgical plans (10). For instance, in cardiovascular surgery, 3D printed models provide detailed displays of congenital heart defects, helping surgeons plan operations and optimize implant positions and sizes (11). In neurosurgery and craniofacial surgery, 3D models offer precise simulations of neurovascular systems and craniofacial bones, aiding in optimal path selection and surgical simulation. These technologies also provide practical training opportunities for surgeons, allowing them to practice surgery on patient-specific anatomical structures in a simulated environment, enhancing operational skills and accuracy (12).

In this study, groups A and B showed shorter surgery durations and less blood loss compared to group C, with statistically significant differences. This result may be attributed to the more intuitive and vivid nature of 3D reconstruction and 3D printing compared to 2D images, helping surgeons make more accurate diagnoses and detailed surgical plans by fully considering potential intraoperative risks. For complex surgeries like ovarian cancer, understanding each patient’s tumor adjacency, blood supply, and relationships with surrounding vital organs preoperatively can mitigate surgical risks, reduce blood loss, and shorten surgery duration. Although group B’s indicators were generally better than group A’s, there were no statistically significant differences between the two groups. This may be due to tumor variability, with differences in tumor spread and diameter even within stage III. Future studies should further refine experimental group stratification to reduce confounding factors.

In postoperative recovery, groups A and B showed significant improvements in time to first flatus, ambulation, and drainage tube placement duration compared to group C, indicating faster recovery for patients using 3D reconstruction and 3D printing technology. We believe this is directly related to shorter surgery durations and less blood loss. However, there were no significant differences in postoperative fever duration and hemoglobin levels on the second postoperative day among the three groups. We consider that timely blood transfusions during surgery with significant blood loss and the extended use of antibiotics postoperatively to suppress inflammatory responses contributed to the lack of significant differences in these indicators.

Regarding postoperative complications and prognosis comparison, statistical results showed no significant differences in postoperative complications and recurrence rates among the three groups (chi-square test, P > 0.05). However, trends in the data suggest that groups A and B exhibited relative advantages in reducing certain postoperative complications. Incidences of hypoalbuminemia, pulmonary infection, and lower limb deep vein thrombosis were relatively lower in groups A and B compared to group C. Although these differences were not statistically significant, they may indicate the potential value of 3D visualization or 3D printing technology in improving surgical planning. These technologies could help surgeons better understand the disease and anatomical structure preoperatively, leading to more appropriate surgical plans and reducing intraoperative trauma and complication risks. Due to statistical non-significance, we cannot ascertain whether these trends reflect the advantages of 3D technology or result from sample size limitations or other unknown factors. Thus, while preliminary data suggests possible benefits for groups A and B in certain aspects over group C, further studies with specific disease classifications and larger sample sizes are needed to verify these observations and clarify the actual effects of 3D technology in reducing complications in ovarian cancer surgery.

Similar studies include Aluwee et al.’s use of MRI-based 3D images and models for preoperative planning, which reduced surgery durations and improved preoperative disease complexity assessments in 10 cases of uterine fibroid resection (13). Teresa Flaxman’s research indicated that 3D models in deep endometriosis surgery cases could help optimize surgeons’ preoperative plans and enhance their ability to visualize complex anatomical structures (11, 12, 14, 15). These cases suggest that utilizing these technologies for preoperative planning can significantly optimize surgical paths, reduce unexpected injuries, and improve surgical safety and success rates.

In conclusion, the application of 3D reconstruction and 3D printing technology in advanced ovarian cancer surgery shows great potential. By providing more accurate preoperative planning, more efficient surgical operations, and faster postoperative recovery, these technologies are expected to improve patient prognosis and survival rates, reduce surgical risks, and increase surgical success rates. In the future, as technology advances and clinical experience accumulates, 3D modeling and 3D printing technology will continue to play a significant role in surgical procedures, bringing better treatment outcomes and quality of life to patients.

## Data Availability

The original contributions presented in the study are included in the article/supplementary material. Further inquiries can be directed to the corresponding author.
